# TLR3 Expression Induces Apoptosis in Human Non-Small-Cell Lung Cancer

**DOI:** 10.3390/ijms21041440

**Published:** 2020-02-20

**Authors:** Francesca Bianchi, Spyridon Alexiadis, Chiara Camisaschi, Mauro Truini, Giovanni Centonze, Massimo Milione, Andrea Balsari, Elda Tagliabue, Lucia Sfondrini

**Affiliations:** 1 Molecular Targeting Unit, Department of Research, Fondazione IRCCS Istituto Nazionale dei Tumori, 20133 Milan, Italy; francesca.bianchi@istitutotumori.mi.it; 2 Pathological Anatomy Unit, ASST Grande Ospedale Metropolitano Niguarda, 20162 Milan, Italy; spyridon.alexiadis@ospedaleniguarda.it (S.A.); mauroa.truini@gmail.com (M.T.); 3 Immunotherapy of Human Tumors Unit, Fondazione IRCCS Istituto Nazionale dei Tumori, 20133 Milan, Italy; chiara.camisaschi@ieo.it; 4 First Pathology Unit, Department of Pathology and Laboratory Medicine, Fondazione IRCCS Istituto Nazionale dei Tumori, 20133 Milan, Italy; giovanni.centonze@istitutotumori.mi.it (G.C.); massimo.milione@istitutotumori.mi.it (M.M.); 5 Dipartimento di Scienze Biomediche per la Salute, Università degli Studi di Milano, 20133 Milan, Italy; andrea.balsari@unimi.it (A.B.); lucia.sfondrini@unimi.it (L.S.)

**Keywords:** toll-like receptor 3 (TLR3), NSCLC, IHC, poly(I:C), apoptosis, CD103, dendritic cells

## Abstract

The prognostic value of Toll-like receptor 3 (TLR3) is debated in cancer, differing between tumor types, methods, and cell types. We recently showed for the first time that TLR3 expression on early stage non-small-cell lung cancer (NSCLC) results associated with a good prognosis. Here, we provide experimental evidences explaining the molecular reason behind TLR3’s favorable prognostic role. We demonstrated that TLR3 activation in vitro induces apoptosis in lung cancer cell lines and, accordingly, that TLR3 expression is associated with caspase-3 activation in adenocarcinoma NSCLC specimens, both evaluated by immunohistochemistry. Moreover, we showed that TLR3 expression on cancer cells contributes to activate the CD103+ lung dendritic cell subset, that is specifically associated with processing of antigens derived from apoptotic cells and their presentation to CD8+ T lymphocytes. These findings point to the relevant role of TLR3 expression on lung cancer cells and support the use of TLR3 agonists in NSCLC patients to re-activate local innate immune response.

## 1. Introduction

To date, major efforts have focused on therapeutic strategies to overcome cancer resistance to immunosurveillance. A successful approach is based on the administration of TLRs ligands to boost antitumor immunity. Toll-like receptors (TLRs) are expressed primarily on immune cells, where they activate downstream signaling cascades that induce the secretion of cytokines and chemokines, culminating in innate and adaptive immune responses against pathogens [1]. Activation of TLRs on professional antigen-presenting cells, such as dendritic cells (DCs), is also crucial for their maturation, their homing into lymph nodes, and to start cytokines production that is necessary for the activation of T-cells [2]. Particularly, various TLR3 agonists are being used in cancer treatments, [3,4,5] and interest in these TLR3 agonists in clinical use as adjuvant for the activation of immune system is constantly growing [6,7,8].

TLR3, like other TLRs, is also expressed on epithelial cells, including cancer cells of several histotypes [9]. Similar to immune cells, cancer cells respond to TLR3 ligands by secreting inflammatory cytokines, type I interferon (IFN I), and chemokines, which enhance the recruitment and activation of immune cells. In addition, TLR3 activation has been reported to mediate apoptosis in several cancer histotypes, primarily through an extrinsic pathway [10]. This unusual function of tumoral TLR3 has increased clinical interest in its targeting, prompting efforts to exploit the direct apoptotic effects of TLR3 on cancer cells [3]. Evidences on hepatocellular carcinoma (HCC), neuroblastomas or esophageal squamous cell carcinoma showed that TLR3 expression by the tumor parenchyma has been associated with a favorable prognosis (reviewed in [11,12]), and we recently observed that in early NSCLC, TLR3 expression on cancer cells is also significantly associated with good overall survival [13].

Our evidence of the prognostic role of TLR3 expression on lung cancer cells reveals the possibility to exploit TLR3 activation to induce cancer cells apoptosis, concurrently with immune activation. Moreover, the induction of apoptosis in lung cancer cells and the release of apoptotic bodies could also contribute to boosting the immune system through immunogenic cell death [14].

Here, we observed that activation of TLR3 causes apoptosis in lung tumor cells in vitro. To determine the relevance of TLR3-induced apoptosis in lung cancer cells in driving good prognosis in NSCLC patients, we explored the expression of cleaved caspase-3 in NSCLC specimens, highlighting that TLR3 expression correlates with cleaved caspase-3 expression in lung adenocarcinoma. Moreover, lung cancer cell death contributed to the activation of CD103+ lung dendritic cells. Collectively, our data indicate that all these TLR3-mediated mechanisms could contribute to explain the association between TLR3 expression on NSCLC tumor cells and a favorable prognosis.

## 2. Results

### 2.1. TLR3 Induces Apoptosis in Lung Tumor Cells

**Figure 1:**
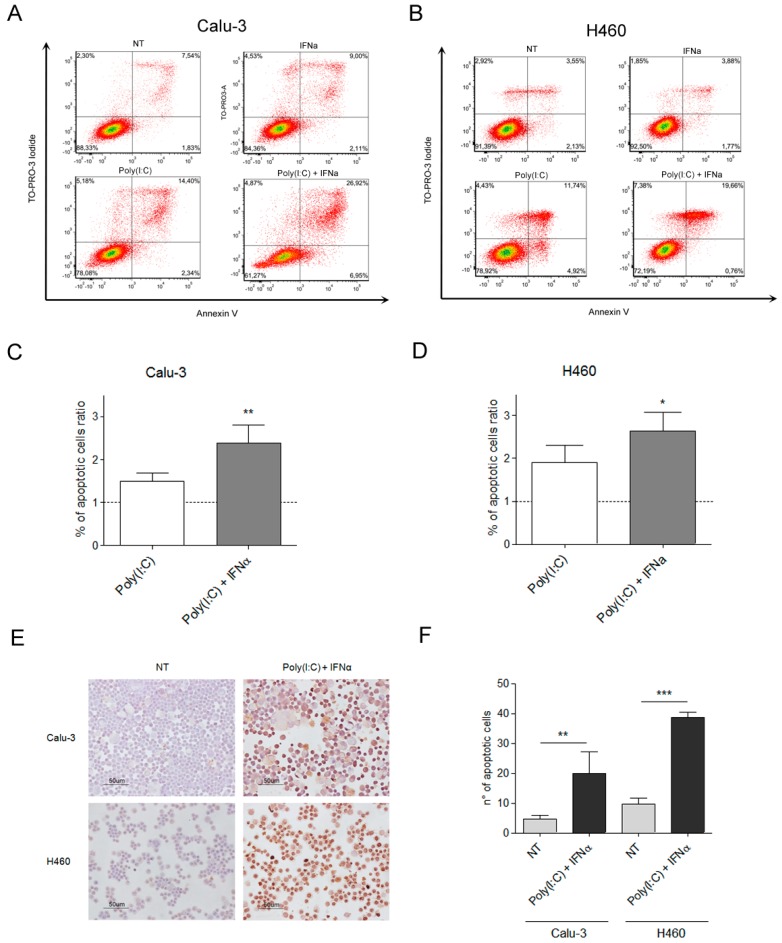
TLR3 activation by Poly(I:C) induces apoptosis in lung cancer cell lines. To determine the percentage of apoptotic cells on TLR3 activation in lung cancer cells, Calu3 (**A**,**C**) and H460 (**B**,**D**) cells were left untreated (NT) or treated with Poly (I:C), IFNα, or their combination for 48 h. Harvested cells were processed by annexin V apoptosis assay. To evaluate the apoptotic cells fractions (expressed as percentage of total number of cells evaluated) of each sample, we considered cells in early and late apoptosis phases, then combining the percentage of cells positive for Annexin V, regardless of TO-PRO-3 staining. Approximately 30% of cells were apoptotic on TLR3 activation in both lung cancer cell lines. The experiment shown in panels A and B is representative of four independent experiments. To calculate the percentage of apoptotic cells ratios, the obtained percentages of total apoptotic cells treated with Poly(I:C)+IFNα were divided for the percentage of total apoptotic cells of the untreated samples, set as reference. The apoptotic ratio increase was confirmed by TUNEL assay (**E**,**F**). * *p* value < 0,05, ** *p* value < 0.005, *** *p* value < 0.001.

Two human lung cancer cell lines that express TLR3 (Figure S1)—Calu-3 (adenocarcinoma) and H460 (large cell carcinoma)—were treated with a synthetic TLR3 agonist, Poly(I:C), to mimic the effects of viral dsRNAs in vitro. Poly(I:C) was combined with IFN type I that is reportedly necessary but not sufficient to activate the apoptotic pathway in melanoma and breast cancer cells [15,16]. Cytofluorimetric annexin V assay showed that around 30% of Calu-3 and of H460 cells die after 48 h incubation with 100 μg/mL Poly(I:C), in combination with 100 U/mL INFα (Figure 1A–D). TUNEL assay confirmed apoptosis in 20% and 40% of Poly(I:C)/INFα-treated Calu-3 and H460 cancer cells, respectively, compared with untreated control cells (Figure 1E,F).

TLR3-mediated mechanism of apoptosis in cancer cells is dependent on caspase-8 activation [10,17], which activates caspase-3 by proteolytic cleavage to amplify caspase-8 apoptosis initiation signals [18]. Consistently, an increase in caspase-3 cleavage was observed in Poly(I:C)/INFα-treated cancer cells (Figure S2).

In Calu-3 and H460 cells, the specific TLR3 agonist Poly(A:U), combined with INFα, induced a 30% increase in the apoptotic cell fraction vs. untreated cells (Figure S3). Since Poly(A:U) is unable to activate MDA5/RIG1, a cytosolic dsRNA recognition receptor alternative to TLR3 that can be bound by Poly(I:C), this data suggests that apoptosis that occurred following treatment with Poly(I:C)/INFα in Calu-3 and H460 cells was related to the specific activation of TLR3.

### 2.2. TLR3 Expression on Cancer Cells Associated with Higher Apoptosis in NSCLC Adenocarcinoma

We examined whether induction of apoptosis in vitro observed upon activation of TLR3 in lung cancer cells also occurs in NSCLC patients with TLR3 expressing tumor. TLR3 protein expression was analyzed in 45 human primary NSCLC specimens that were collected at ASST Grande Ospedale Metropolitano Niguarda. Median age was 65.5 years, 67% were male and 76% were adenocarcinoma (Table S1).

**Figure 2:**
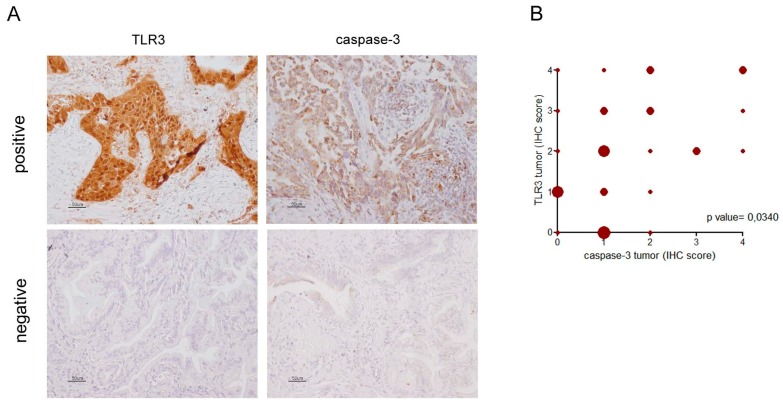
TLR3 and caspase-3 immunohistochemical expression in NSCLC. TLR3 and caspase-3 immunohistochemical staining were performed on FFPE NSCLC sections as described in Materials and Methods. TLR3 and caspase-3 expression was defined as the percentage of positive cells of the total number of cancer cells in the sample in tumor (IHC score: 0= no positive cells; 1 ≤ 25%; 25% < 2 ≤ 50%; 50% < 3 ≤75%; 4 > 75%). (**A**) Images of cases positive and negative both for TLR3 and caspase-3 in the tumor cells. Images are acquired at 20× magnification. (**B**) Correlation analysis between percentage of IHC-positive cells for TLR3 expression with percentage of IHC-positive cells for caspase-3 expression in the tumor in adenocarcinoma NSCLC (*n* = 33; *p* = 0,0365; Pearson *r* = 0,3655; 95% confidence interval 0.02532 to 0.6299). Shape size is related to the number of cases represented.

IHC performed on FFPE NSCLC specimens showed TLR3 and caspase-3 expression in tumor cells (Figure 2A). TLR3 and caspase-3 expression were scored as described in Materials and Methods section. Considering all cases, no correlation emerged between TLR3 and caspase-3 expression in tumor cells; whereas, by considering only lung adenocarcinoma, a significant direct correlation was observed (Figure 2B; *p* = 0.0340, Pearson test; *n* = 33). Indeed, cases with an elevated percentage of tumor cells expressing caspase-3 showed concurrently a high percentage of tumor cells expressing TLR3 (>50% of total tumor cells; score 2, 3, 4); conversely, most of the cases (11/13 cases, 85%) with TLR3 low expression on tumor cells showed a concomitant low expression of caspase-3 on tumor cells (≤25% of total tumor cells; score 0, 1).

**Figure 3:**
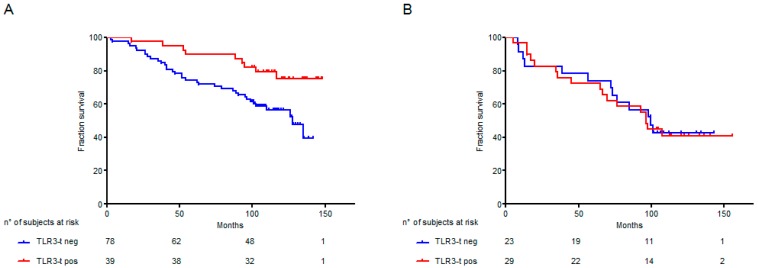
Kaplan–Meier plots of Overall Survival (OS) according to NSCLC histotype and TLR3 immunohistochemistry expression. The relationship between TLR3 expression and prognosis, according to NSCLC histotype, was examined in a cohort of NSCLC specimens, collected at Fondazione IRCCS Istituto Nazionale Tumori Milan [13]. NSCLC cases were considered positive for TLR3 expression (TLR3-t) with a percentage of positive tumor cells >50%. (**A**) Kaplan–Meier plots of OS of 117 adenocarcinoma NSCLC patients stratified according to TLR3-t immunohistochemistry expression; (**B**) Kaplan–Meier plots of OS of 52 squamous NSCLC patients stratified according to TLR3-t immunohistochemistry expression; Red line: cases positive for TLR3-t expression; blue line: cases negative for TLR3-t expression.

We previously analyzed a cohort of 194 human primary NSCLC specimens (who had been diagnosed between 2003 and 2007 at our institute Fondazione IRCCS Istituto Nazionale Tumori Milan, selected all stage I, with a median follow-up time of 105.7 months) [13], showing a significant association between prognosis (Overall Survival, OS) and TLR3 tumor expression. Here, a new association analysis between OS and TLR3 immunohistochemistry expression on tumor cells has been explored according with NSCLC histology. TLR3 expression was significantly associated with prognosis strongly in subjects with adenocarcinoma (*p* = 0.0117; HR = 0.389, Cl = 0.187–0.811; *n* = 117) (Figure 3A), whereas no significant association between TLR3 expression on tumor cells and prognosis was observed considering only squamous carcinoma (*p* = 0.8826; HR = 1.056, Cl = 0.512–2.176; *n* = 52) (Figure 3B).

**Figure 4:**
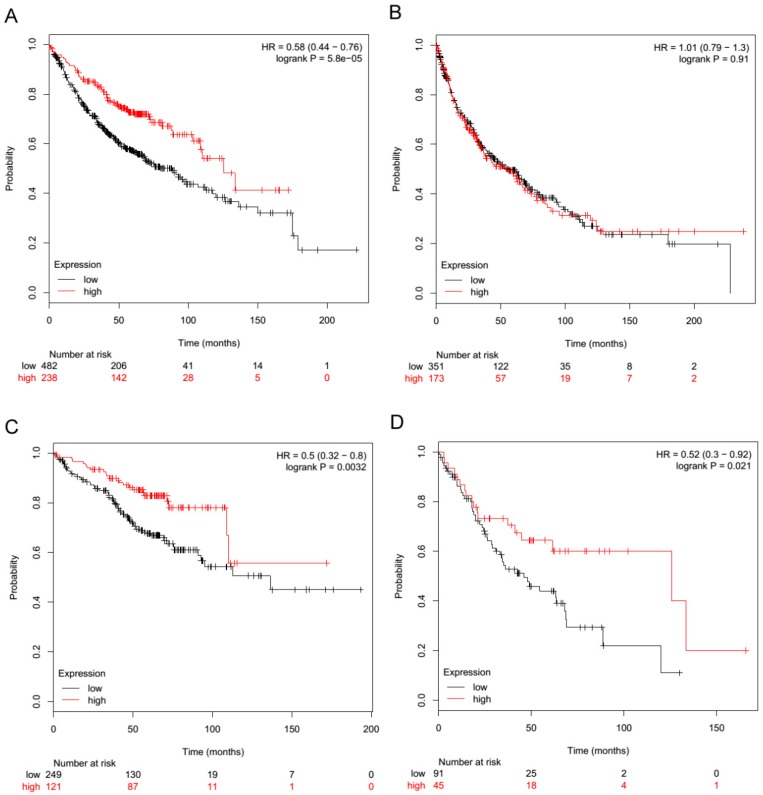
TLR3 mRNA expression is associated with a good prognosis in adenocarcinoma NSCLC. The relationship between TLR3 expression and prognosis was examined in the KM-Plotter public gene expression NSCLC datasets [19]. NSCLC patients were stratified by tertiles with regard to TLR3 mRNA expression (probe ID 206271_at). Red line: high TLR3 expression; black line: low TLR3 expression. (**A**) Overall survival probability of patients by TLR3 mRNA levels in adenocarcinoma NSCLC cases, *n* = 720. (**B**) Overall survival probability of patients by TLR3 mRNA levels in squamous NSCLC cases, *n* = 524. (**C**) Overall survival probability of patients by TLR3 mRNA levels in only stage I adenocarcinoma NSCLC cases, *n* = 370. (**D**) Overall survival probability of patients by TLR3 mRNA levels in only stage II adenocarcinoma NSCLC cases, *n* = 136.

Moreover, by in silico analysis of NSCLC patients in the KM-Plotter public NSCLC gene expression dataset [19] according with TLR3 expression, the histotype of NSCLC resulted in being an important determinant for the association between TLR3 and good prognosis. Indeed, TLR3 expression was significantly associated with a good prognosis in adenocarcinoma (Figure 4A; *p* < 0.001; HR = 0.58; *n* = 720) but not in squamous carcinoma (Figure 4B; *p* = 0.91; HR = 1.01; *n* = 524). According with tumor stage, we observed that both in stage I and stage II adenocarcinoma, TLR3 was significantly associated with a good prognosis (Figure 4C; *p* = 0.0032; HR = 0.5; *n* = 370) (Figure 4D; *p* = 0.021; HR = 0.52; *n* = 136). In agreement, a significantly higher time to first progression (FP) was observed in NLSLC patients expressing TLR3 mRNA, highlighting the association between TLR3 expression and recurrence (Supplementary Figure S4).

Moreover, a correlation between TLR3 and caspase-3 mRNA expression in adenocarcinoma patients was investigated through the multiple gene analysis options of the KM-Plotter public NSCLC gene expression dataset. A light correlation between TLR3 and caspase-3 mRNA expression was observed in adenocarcinoma patients (all cases, Spearman coefficient 0.5682; only stage I, Spearman coefficient 0.6415).

These data support the role of TLR3 expression in inducing apoptosis in lung adenocarcinoma cells.

### 2.3. TLR3 Boosts the Innate Immune Response

We explore the possibility that TLR3-mediated apoptosis activates immune cells against the tumor. Of the various populations of lung dendritic cells (DCs), the CD103+ subset is specifically deputed in the lung to process antigens from apoptotic cells and to present them to CD8+ T lymphocytes, inducing the activation of adaptive immune cells [20]. Thus, we determined whether the induction of apoptosis in lung tumor cells by TLR3 agonist treatment specifically activates the CD103+ cell subpopulation. An in vitro co-culture of lung-infiltrating immune cells, derived from immunocompetent mice and TLR3-positive tumor cells that were pretreated with Poly(I:C)/INFα, was established (Figure S3 and Figure S4 describe the experimental flowchart and gating strategy, respectively). Expression of cell surface markers CD80, CD86 and CD83 have been assessed to evaluate the percentage of activated immune cells.

**Figure 5:**
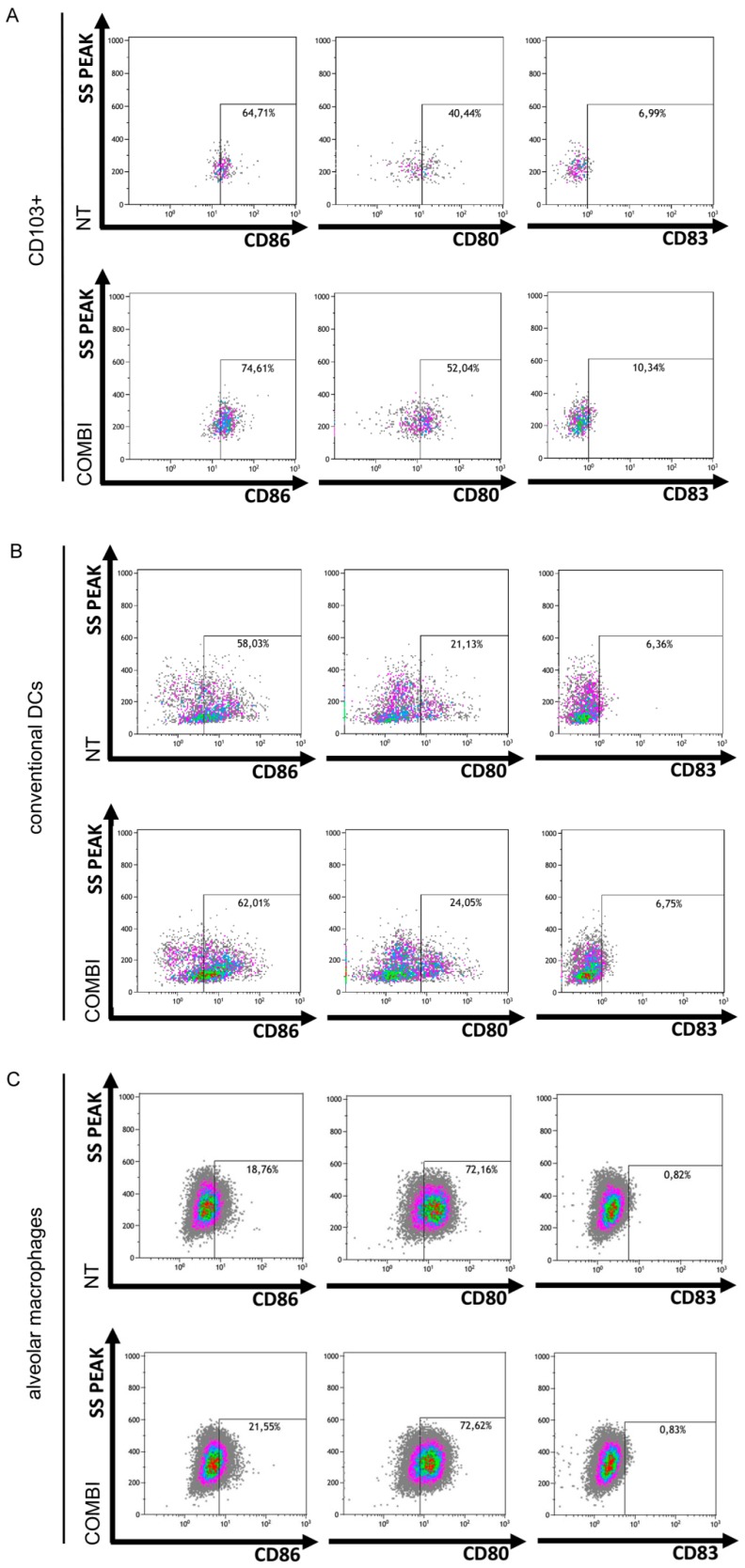
Lung cancer cell apoptosis mediated by TLR3 induces in vitro CD103+ lung DC activation. Murine lung immune cells were co-cultured with H460 cells that were untreated (NT) or pretreated with Poly(I:C)/INFα (COMBI), as in Figure S5. CD103+ DCs (**A**), conventional DCs (CD11b+ DCs) (**B**) and alveolar macrophages (AMs) (**C**) were identified by cytofluorimetric analysis using the gating strategy reported in Figure S6. The percentage of CD86, CD80, and CD83 expression in CD103+ DCs (A), conventional DCs (B) and AMs (C) was determined and reported as dot plots in figure, comparing NT vs. COMBI.

The total number of activated CD103+ CD86+ and of CD103+ CD80+ cell subsets rose 10% and 12% by FACS, respectively, in the immune cells co-cultured with Poly(I:C)/INFα-treated-H460 cancer cells versus untreated H460 cells (Figure 5A). CD103+ CD83+ cells increased by 4% (Figure 5A). A similar increase in the CD103+ fractions, which were positive for CD80, CD83 and CD86 activation markers, was observed in Calu-3 cells (Figure S7). Higher expression of these markers, albeit to a lesser extent, was also observed in conventional dendritic cells (CD11b+ CD11c+ CD103− DCs) (Figure 5B) (+4% CD86 and +3% CD80), but CD86, CD83, and CD80 were not upregulated in alveolar macrophages (Figure 5C).

These findings indicate that the in vitro stimulation of TLR3-positive human lung cancer cells with synthetic TLR3 agonist induces apoptosis in tumor cells and that TLR3-mediated apoptosis of lung cancer cells increases the activation of the CD103+ subset of lung dendritic cells.

## 3. Discussion

We and others observed the expression of TLR3 protein NSCLC specimens by immunohistochemistry [13,21], and we revealed that the expression of TLR3 on tumor cells has a favorable prognostic value in early stage human NSCLC [13]. Here, our evidences indicate the promotion of apoptosis cascade and the engagement of the immune system as the two main outcomes of TLR3 activation on tumor cells. The presence of exogenous or endogenous ligands, inhaled or released from dying tumor cells, might sustain TLR3 activation, thus explaining the improved prognosis of NSCLC patients with high TLR3 expression in tumor cells. In agreement with this hypothesis, in this study we observed that specific stimulation of TLR3 with synthetic TLR3 agonists and INFα is able to induce cell apoptotic program, culminating in the activation of caspase-3 in two human lung cancer cell lines expressing TLR3. Furthermore, we also observed for the first time that TLR3 expression on cancer cells is significantly associated with cleaved caspase-3 expression in a cohort of 45 human primary adenocarcinoma NSCLC. The association between TLR3 and caspase-3 expression, as a proof of the activation of apoptosis cascade in lung cancer, is supported by evidences in other cellular models of different cancer types, including breast, melanoma, head and neck, prostate, renal carcinoma, colon, and cervical cancers [10,11].

Here, we then showed that TLR3-mediated apoptosis of lung cancer cells indirectly contributes to the activation of immune response against tumors. We showed that apoptotic bodies from dying tumor cells stimulate APCs, rendering them more efficient with regard to antigen presentation [22]. In Calu-3 and H460 cells, TLR3-induced apoptosis primarily activates lung CD103+ DCs, a subset of pulmonary DCs that preferentially acquires and presents apoptotic cell-associated antigens [20]. The capability of TLR3 agonists to boost the immune response in tumor microenvironment has been widely investigated in preclinical models [23]. TLR3 stimulation triggers DC maturation through expression of co-stimulatory molecules, leading to NK cells activation and promotion of cross-priming that result in the CTLs induction [24,25]. Moreover, Poly(I:C) can in vivo convert lung tumor-associated macrophages (TAM) from tumor supporters (M2) to those with tumoricidal properties (M1) [26]. Thus, besides these effects of TLR3 activation on immune cells, our data indicate that TLR3 activation on cancer cells by promoting apoptosis may in turn favor the activation of an immune response against the tumor and thus improve the prognosis of lung cancer patients.

Considering all cases of NSCLC collected at Niguarda Hospital, no correlation emerged between TLR3 and caspase-3 expression in tumor cells, whereas, by considering only lung adenocarcinomas, a significant direct correlation was observed. Accordingly, when we analyzed the human primary NSCLC specimens collected at Fondazione IRCCS Istituto Nazionale dei Tumori of Milan, which we recently investigated for TLR3 expression [13], a benefit from TLR3 tumor expression was observed only in subjects with adenocarcinoma, but not in patients with squamous carcinoma. We can speculate that the antitumor effect of TLR3 activation on tumor cells depends on the histological characteristics of the NSCLCs and the environmental immune context in which these tumors develop. In agreement, Jiang et al. [27], based on the analysis of a set of immunological markers, reported that squamous NSCLC is characterized by divergent clinical and molecular immune phenotypes compared with non-squamous NSCLC. As a support of the relevance of NSCLC histotype to consider the TLR3 prognostic role, the in silico analysis of KM-Plotter database showed a better outcome in TLR3 expressing adenocarcinoma and no association between TLR3 expression and prognosis in squamous NSCLC, both considering stage I and stage II NSCLC patients. A deeper characterization of the complex and bidirectional cross-talk between TLR3-expressing cancer cells and immune populations is thus required to define TLR3 feasibility both as a prognostic marker and therapeutic target.

The method of TLRs’ agonists delivery still remains the major limitation to their clinical use, since they exploit a major action when locally dosed, accordingly to the importance of innate effector cell activation at the site of tumor growth [28]. A strategy we are currently evaluating to improve the efficacy of TLRs agonists is the local administration. Indeed, we recently demonstrated that aerosol administration of TLR3 agonist poly(I:C) in mouse models impaired lung metastatization through activation of TLRs on lung cancer infiltrating immune cells [29]. Particularly, aerosolization of CpG-ODN with Poly(I:C) into the bronchoalveolar space reduced the presence of M2-associated arginase- and IL-10-secreting macrophages in tumor-bearing lungs and increased the antitumor activity of aerosolized CpG-ODN alone against B16 lung metastases. This effect was due to an enhanced recruitment and cytotoxic activity of tumor-infiltrating NK cells in the lung mediated by alveolar macrophages [30].

Therefore, local administration of TLR3 agonists in NSCLC patients could lead to the re-activation of local innate immune response and apoptosis of lung adenocarcinoma cells.

## 4. Materials and Methods

### 4.1. Cell Lines and Treatments

The human lung cancer cell lines Calu-3 and H460 were maintained at 37 °C in a humidified atmosphere of 5% CO2 in air in RPMI 1640 (Life Technologies) supplemented with 10% fetal bovine serum (FBS) and 2 mM glutamine (both from Life Technologies). For indicated treatment, the Calu-3 and H460 human lung cancer cell lines were seeded in 6-well plates and treated with 100 U/mL INFα, as it is standard for determining the in vitro effects of TLR3 agonists [15]. After 24 h, the culture medium was replaced with 100 μg/mL Poly (I:C) (LMW) (tlrl-picw, InvivoGen), or Poly(A:U) 500 μg/mL (tlrl-pau, InvivoGen) and 100 U/mL INFα for additional 48 h.

### 4.2. Quantitative PCR Analysis

RNA was isolated using QIAzol (QIAGEN), according to the manufacturer’s instructions from lung cancer cells. Reverse transcription was performed as we already described [31] using a High-Capacity RNA-tocDNA Kit (Applied Biosystems-Thermo Fisher Scientific). Real-time PCR was performed using TaqMan^®^ Fast Universal PCR Master Mix (Applied Biosystems-Thermo Fisher Scientific) and SDS 2.4 on a 7900HT Fast Real-Time PCR System (Applied Biosystems-Thermo Fisher Scientific). The TaqMan^®^ gene expression assays for TLR3 used was Hs01551078_m1 (Applied Biosystems-Thermo Fisher Scientific). The expression of TLR3 gene was normalized to β2m (assay ID: Mm00437762_m1). PCR data were analyzed using the 2^−ΔCt^ method.

### 4.3. Cytofluorimetric Analysis of Lung Cancer Cell Apoptosis

The Calu-3 and H460 human lung cancer cell lines were left untreated or treated with 100 U/mL INFα and after 24 h, the culture medium was replaced or not with 100 μg/mL Poly (I:C) (LMW) (tlrl-picw, InvivoGen) for an additional 48 h. At the end of treatment, the supernatants from tumor cells, containing debris, necrotic cells, and trypsin-detached cells, were centrifuged for 5 min at 1500 rpm. The recovered cells were washed in 1× PBS. Cytofluorimetric analyses were performed with the Flow Cytometry service of IRCCS National Cancer Institute, Milan. To evaluate apoptosis by annexin V assay, cells were processed using the FITC Annexin V Apoptosis Detection Kit I (BD Pharmigen) and TO-PRO-3 (1:2000, Thermo Fisher Scientific) following the manufacturer’s instructions. The samples were processed on a FACSCanto (BD Biosciences), and the data were analyzed using FlowJo (TreeStar). In each sample, the amount of viable cells in early apoptosis, late apoptosis, and necrosis was expressed as the percentage of total cells. To evaluate the apoptotic cells fractions (expressed as percentage of total number of cells evaluated) of each sample, we considered cells in early and late apoptosis phases, then combining the percentage of cells positive for Annexin V, regardless of TO-PRO-3 staining. To calculate the percentage of apoptotic cells’ ratio, the obtained percentage of total apoptotic cells treated with Poly(I:C)+IFNα were divided for the percentage of total apoptotic cells of the untreated samples, set as reference. The same procedure was followed for the induction of apoptosis by Poly(A:U) 500 μg/mL in lung cancer cell lines.

### 4.4. TUNEL Assay

The Calu-3 and H460 human lung cancer cell lines were seeded in 6-well plates and treated with 100 U/mL INFα, as it is standard for determining the in vitro effects of TLR3 agonists [15]. After 24 h, the culture medium was replaced with 100 μg/mL Poly (I:C) (LMW) (tlrl-picw, InvivoGen) and 100 U/mL INFα. After an additional 48 h, the supernatants from tumor cells, containing debris, necrotic cells, and trypsin-detached cells, were centrifuged for 5 min at 1500 rpm. The recovered cells were washed in 1X PBS. For the TUNEL assay, cytospins were settled: Recovered cells were placed on Superfrost slides (Thermo Fisher Scientific) on a Thermo Shandon Cytospin 3 centrifuge and fixed with paraformaldehyde 4%, pH 7.4. The cytospins were processed using the in situ Cell Death Detection Kit-POD (Roche) following the manufacturer’s instructions.

### 4.5. Cytofluorometric Analysis of Caspase-3 Activation

Calu-3 cells were untreated or treated with a combination of 100 U/mL INFα and 100 μg/mL Poly(I:C). After 48 h, the cells were stained with FITC Rabbit Anti-Active Caspase-3 (CPP32; Yama; Apopain) (BD Pharmingen, cat. 5168654X) and propidium iodide (PI), and the percentage of cells that were positive for caspase-3 was determined by FACS analysis. The samples were analyzed on a FACSCanto (BD Biosciences), and the data were processed using FlowJo software (TreeStar).

### 4.6. Patients

Samples from 45 NSCLC patients who had been diagnosed between 2009 and 2011 at ASST Grande Ospedale Metropolitano Niguarda were selected. The study was conducted in accordance with the Declaration of Helsinki (World Medical Association, 2013), and the study was conducted after the approval from the Niguarda Administrative Authority and the Independent Ethics Committee of Milan Area 3 (Milan, Italy) (No. 253-062019).

### 4.7. Immunohistochemical Analysis of FFPE NSCLC Specimens

TLR3 and caspase-3 were analyzed by IHC on 2.5/3-µm formalin-fixed, paraffin-embedded (FFPE) tumor sections, using the polyclonal anti-TLR3 clone 40F9.6 at 1:1000, which was developed by Salaun et al. [32] and kindly provided by Innate Pharma (Innate Pharma, Marseille France), and the monoclonal Cleaved Caspase-3 (Asp175) (5A1E) #9664 at 1:150. Briefly, for TLR3 staining, antigen retrieval was performed by heating the slides for 10 min at 96 °C in EDTA 1mM pH 8; immunoreactions were visualized using streptavidin-biotin-peroxidase (Thermo Fisher Scientific), 3,3′-diaminobenzidine (DAB; brown signal) (Dako) as the chromogen. For caspase-3 staining, antigen retrieval was performed by heating the slides for 10 min at 100 °C in citrate buffer pH 6 overnight; immunoreactions were visualized using Novolink polymer system (Leica). The sections were counterstained with hematoxylin, and immunoreactions were visualized and acquired as we already described [33,34]. The reactivity of anti-TLR3 and of anti-caspase-3 in the IHC analysis of FFPE NSCLC specimens was scored as the percentage of positive cells of the total number of cancer cells present in the tumor sample. TLR3 and caspase-3 expression was defined in tumor cells using a semiquantitative method that defined the percentage of positive cells of the total number of cancer cells in the sample (0 = no positive cells; 1 ≤ 25%; 25% < 2 ≤ 50%; 50% < 3 ≤ 75%; 4 > 75%). Cases expressing TLR3 >50% of the total number of cancer cells were compared with the remaining cases expressing TLR3 ≤50% of the total number of cancer cells. The distribution of cases by expression pattern is shown in Figure S8.

### 4.8. TLR3 Expression in NSCLC by Gene Expression Microarray

TLR3 expression was assessed in a large meta-analysis of NSCLC datasets on an Affymetrix platform [19,35], as we already described for other cancer types [36]. Briefly, the online KM-Plotter database, which includes information on 22,277 genes and their influence on survival in 1926 NSCLC patients, was used for the survival analysis. NSCLC patients were stratified by tertiles of TLR3 mRNA expression (probe ID 206271_at). The overall survival probability of patients by TLR3 mRNA expression was calculated for adenocarcinoma NSCLC cases (*n* = 720), squamous NSCLC cases (*n* = 524), stage I adenocarcinoma NSCLC cases (*n* = 370), and stage II adenocarcinoma NSCLC cases (*n* = 136).

### 4.9. Immunofluorescence Analysis of Mouse Lung Immune Infiltrates

Lung immune cells from the lungs of treated healthy mice were isolated as described [30]. Cell suspensions were stained directly for flow cytometry. Briefly, mouse lungs were homogenized mechanically in DMEM medium that was supplemented with 10% fetal bovine serum (FBS), centrifuged for 5 min at 1500 rpm and 4 °C, and digested in DMEM medium that contained 300 U/mL collagenase and 100 U/mL hyaluronidase (Stemcell Technologies, 07912) for 1 h at 37 °C. Cell suspensions were filtered through 40-µm cell strainers, and red blood cells were lysed in ACK (BioWhittaker LONZA) for 10 min on ice. Next, the samples were centrifuged for 5 min at 1500 rpm at 4 °C and washed with 1× PBS.

Co-cultures were established in complete medium (RPMI 10% FBS, Na pyruvate 1:100, Hepes 1:100, glutamine 1:100, and penstrep 1:100, Thermo Fisher Scientific) by incubating 500,000 cancer cells (Calu-3 or H460) that were pretreated with Poly (I:C) and INFα with 2,000,000 cells that had been recovered from mouse lungs for 1.5 h or 4 h at 37 °C with gentle rocking. At the end of the incubation, the cells were centrifuged for 5 min at 1500 rpm and washed in 1× PBS for FACS analysis.

Immunofluorescence staining was performed as already described [34]. Briefly, the cells were labeled using the Live/Dead Fixable Violet Dead Cell Stain Kit (Life Technologies) for 30 min at 4 °C in the dark, washed with 1× PBS, and stained for 30 min at 4 °C with the following directly conjugated antibodies: anti-mouse CD11b Alexa Fluor 700, anti-mouse CD11c PE-Cy7, anti-mouse CD86 PE (Milteny), hamster anti-mouse CD80 PE-CF594, rat anti-mouse CD83 BV510 (BD Horizon), anti-mouse MHCII PERcP Vio700, and anti-mouse CD103 APC (Milteny). A purified rat anti-mouse CD16/CD32 MAb (eBiosciences, clone 93) was used to block nonspecific binding to mouse Fc receptors. Fluorescence compensation was performed using CompBeads Anti-Rat, Anti-Hamster Ig, CompBeads Negative Control (BD Biosciences), ArC reactive beads, and ArC negative beads (Life Technologies). The cells were acquired with a Gallios flow cytometer (Beckman Coulter) and analyzed using FlowJo (TreeStar) or Kaluza software (Beckman Coulter) with the gating strategy reported in Figure S6. Each experiments has been performed at least two times, and a representative one is shown.

### 4.10. Statistical Analysis

In in vitro studies, data were expressed as mean ± standard deviation. Differences were tested by two-tailed *t*-test; for two dependent groups, the equality of means was examined by two-tailed paired t-test. Pearson’s correlation was used to analyze the association between TLR3 and caspase-3 expression in the Niguarda cohort. The values *p* < 0.05 were considered statistically significant. In the INT cohort, OS was defined as the time between the date of surgery and the date of death from any cause or the date of the last follow-up. Univariate survival analysis was carried out by phreg procedure using a Cox regression model and the determination of the statistical significance of all categorical predictors by chi-square test. The effects of explanatory variables on event hazard were quantified by hazard ratios (HR), and 95% confidence limits (Cl) are indicated [37]. All statistical analyses were conducted using SAS (SAS 9.4 Institute Inc., Cary, NC, USA).

## Supplementary Materials

Supplementary materials can be found at https://www.mdpi.com/1422-0067/21/4/1440/s1.
