# Supplementary material for: TLR3 Expression Induces Apoptosis in Human Non-Small-Cell Lung Cancer
Source: Int J Mol Sci. 2020 Feb 20;21(4):1440. doi: 10.3390/ijms21041440 (PMC7073031; doi:10.3390/ijms21041440)
Supplement: Supplementary file 1 [file ijms-21-01440-s001.pdf]

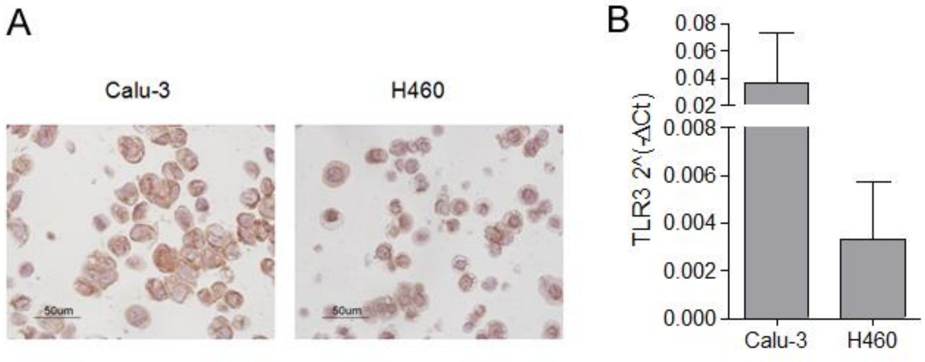

Supplementary Fig. 1. TLR3 protein and mRNA levels in Calu-3 and H460 lung cancer cell line. A) TLR3 protein expression was determined on FFPE section of Calu-3 and H460 cyto-included cells. B) TLR3 mRNA level was determined in Calu-3 and H460 cells by Real-time PCR by using TaqMan assay for TLR3 and normalized on TaqMan assay for β2m.

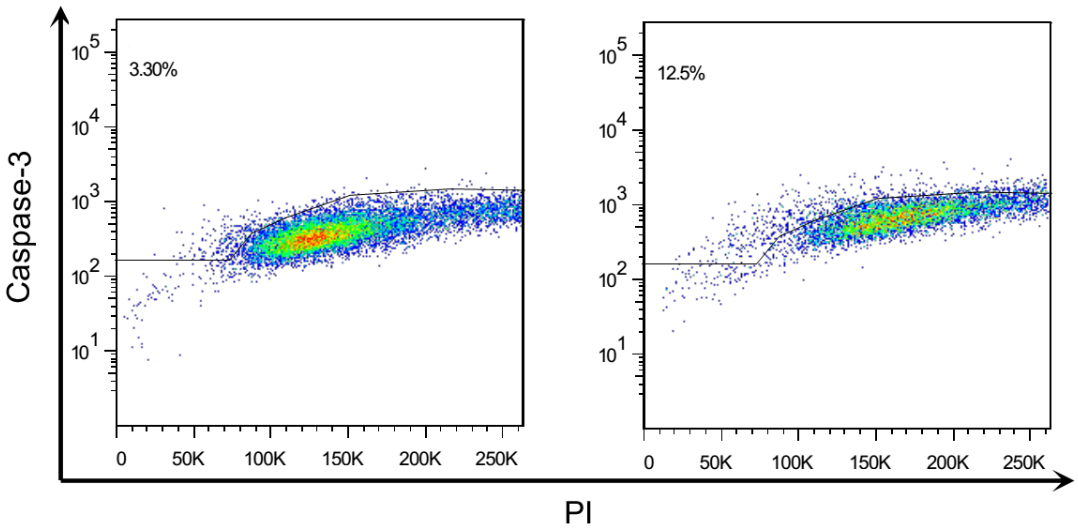

Supplementary Fig. 2. TLR3 activation induces caspase-3 cleavage in Calu-3 lung cancer cell line. Calu-3 cells were left untreated or treated with a combination of INFα and Poly(I:C). After 48 hr cells were stained with FITC Rabbit Anti-Active Caspase-3 antibody (CPP32; Yama; Apopain) (BD Pharmingen, cat. 5168654X) and propidium iodide (PI) following manufacturer's instructions. The percentage of cells positive for activated caspase-3 was determined by FACS analysis.

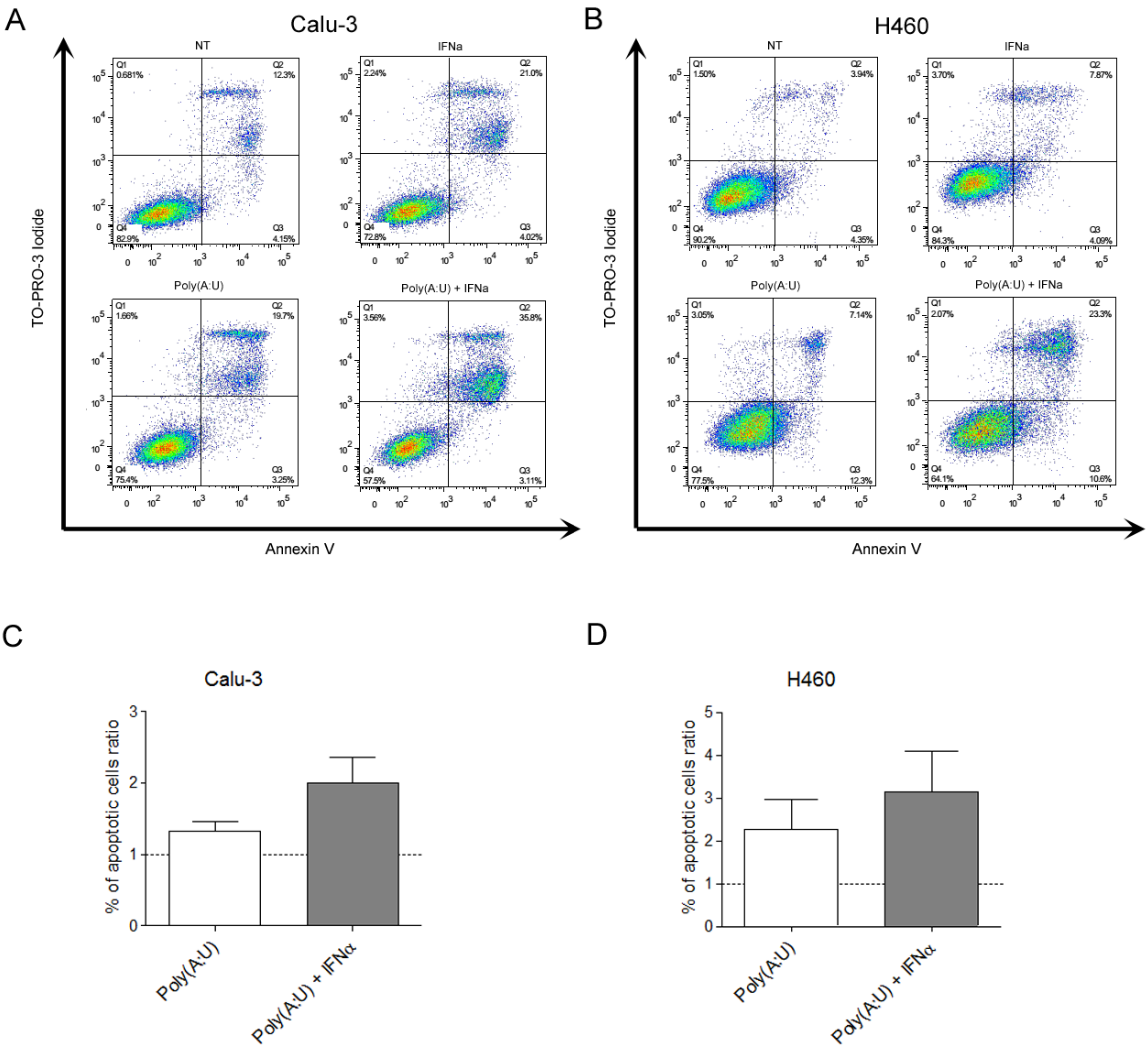

Supplementary Fig. 3. TLR3 specific activation by Poly(A:U) induces apoptosis in lung cancer cell lines. Calu-3 (A, C) and H460 (B, D) cells were left untreated (NT) or treated 48 h with IFNα (100 UI/ml), Poly(A:U) (500µg/ml) or a combination of both. The percentage of apoptotic cells was determined by FACS analysis of Annexin V assay. The experiment shown in panels A and B is representative of 2 independent experiments.

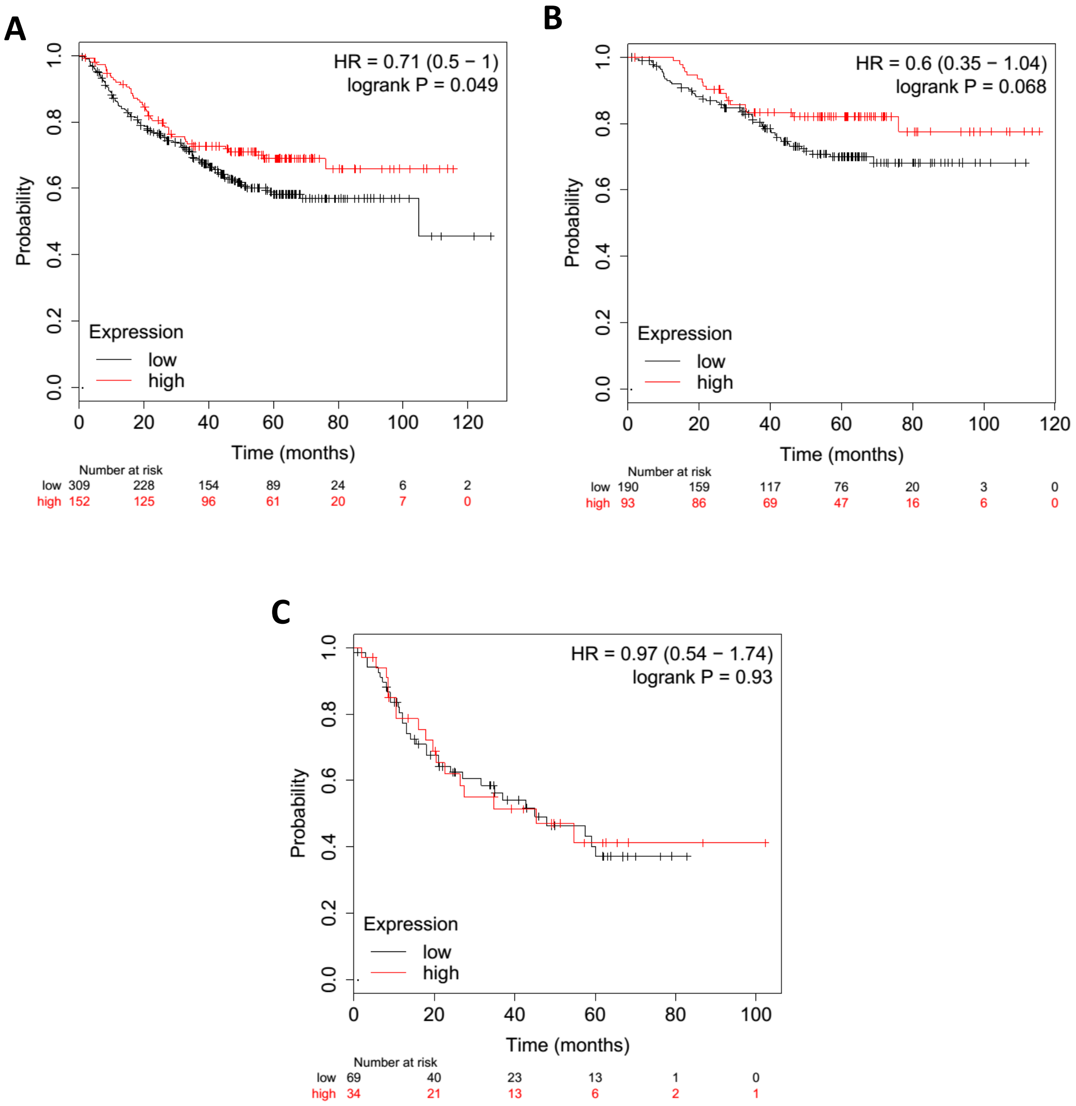

Supplementary Fig. 4. TLR3 mRNA expression is associated with a time to progression in adenocarcinoma NSCLC. The relationship between TLR3 expression and time to progression was examined in the KM-Plotter public gene expression NSCLC datasets [19]. NSCLC patients were stratified by tertiles with regard to TLR3 mRNA expression (probe ID 206271_at). Red line: high TLR3 expression; black line: low TLR3 expression. A) First progression survival probability of patients by TLR3 mRNA levels in adenocarcinoma NSCLC cases, n= 461. B) First progression survival probability of patients by TLR3 mRNA levels in stage I adenocarcinoma NSCLC cases, n= 283. C) First progression survival probability of patients by TLR3 mRNA levels in stage II adenocarcinoma NSCLC cases, n= 103.

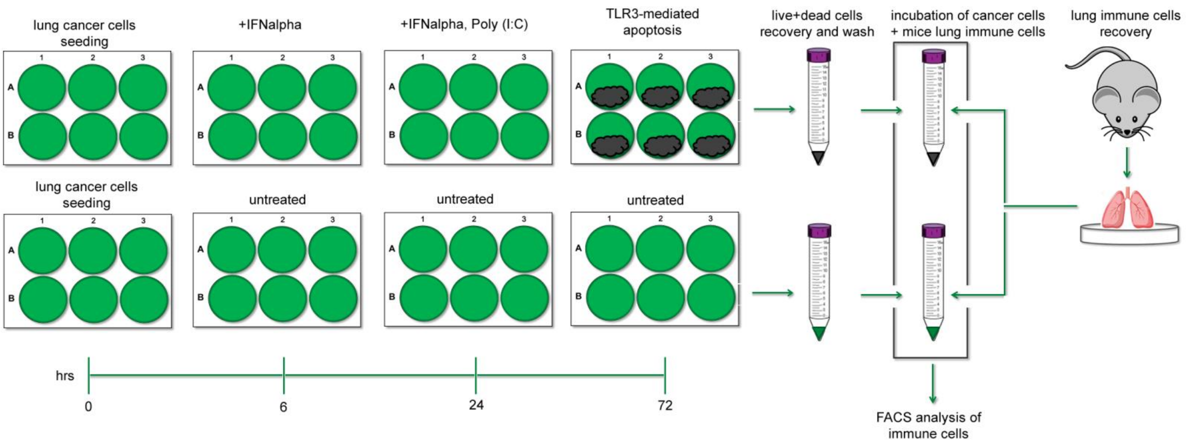

Supplementary Fig. 5. Flow-chart of lung cancer cells and immune infiltrate co-culture experiment. Co-culture was performed by incubating 500,000 tumor cells (Calu-3 or H460) pretreated with Poly(I:C) [100 ug/ml] + INFα [600 U/ml] (as described in Material and Methods) with 2,000,000 pulmonary infiltrate cell derived ex-vivo from immunocompetent mice (as described in Material and Methods) in complete medium (RPMI + 10% FBS, Na Pyruvate 1: 100, Hepes 1: 100, Glutamine 1: 100 and Penn-strep 1: 100) in conical PET tube (Falcon) for 4 hours at 37 °C.

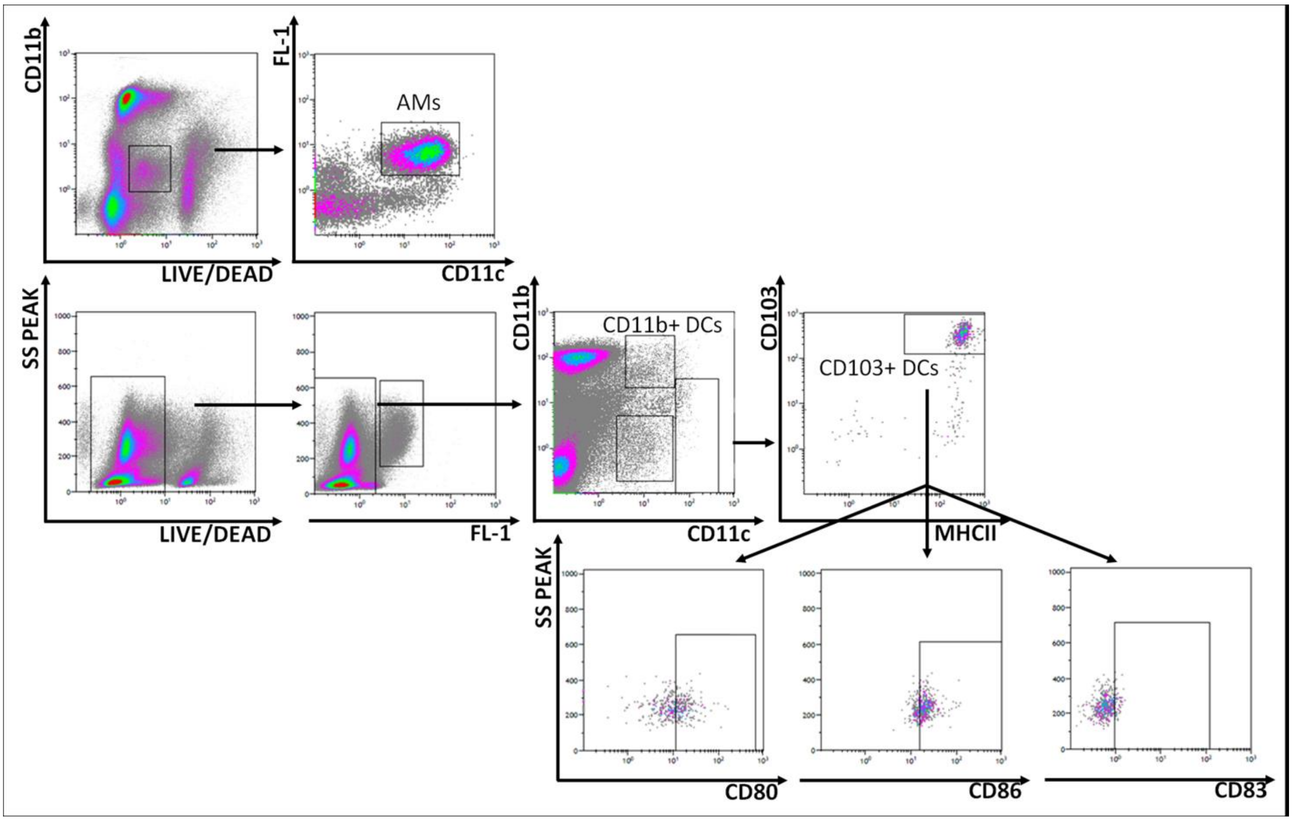

Supplementary Fig. 6. Gating strategies used to analyze lung suspensions. Alveolar macrophages (AMs), were identified as CD11c+FL-1+ cells among live CD11blow cells after doublet cell exclusion (FL-1 channel was reserved for the assessment of autofluorescence); CD103+ DCs were identified as CD103+MHCII^{high} inside CD11c^{high}CD11b^{low} cells gated among live FL-1- cells after doublet cell exclusion; conventional DCs (CD11b+ DCs) were identified as CD11b+CD11c+ cells gated among live FL-1- cells after doublet cell exclusion. The latter dot plots show a representative example of CD80, CD86 and CD83 expression among CD11b+ DCs gated as above.

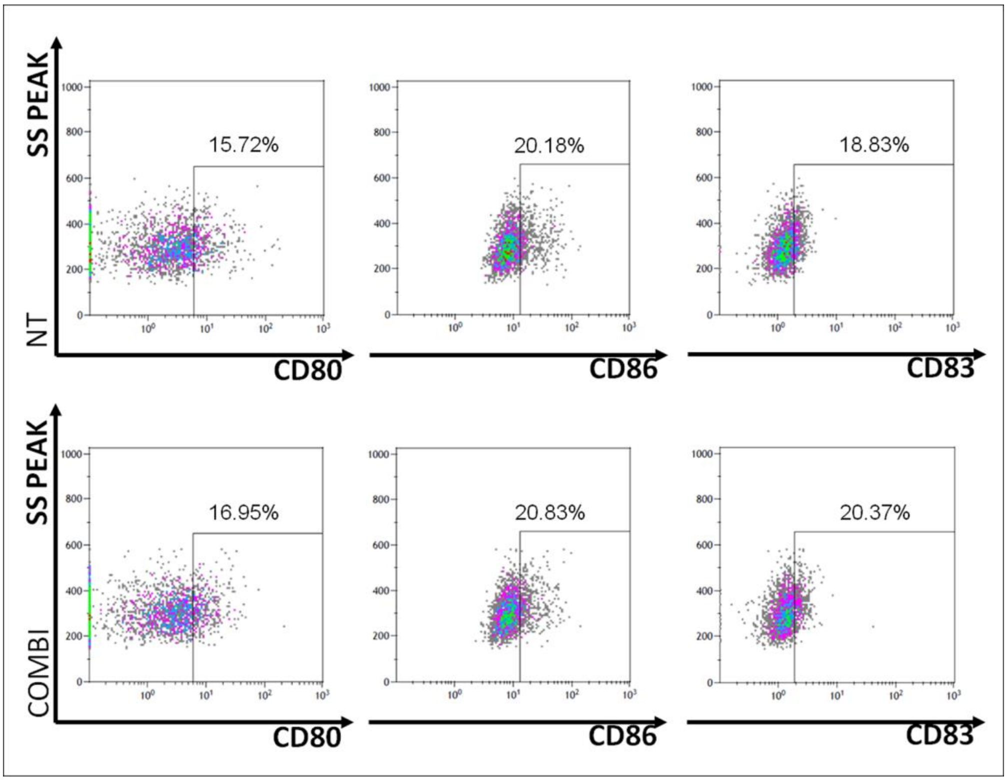

Supplementary Fig. 7. Analysis of CD103+ lung DC activation on i*n vitro* co-culture with TLR3-mediated apoptotic Calu-3 cells. Murine lung immune cells were co-cultured with Calu-3 cells that were untreated (NT) or pretreated with Poly(I:C)/INFα (COMBI) as in Supplementary Fig. 5. CD103+ DCs were identified by cytofluorimetric analysis using the gating strategy reported in Supplementary Fig. 6. The percentage of CD86, CD80, and CD83 expression in CD103+ DCs was determined and reported as dot plots in figure comparing NT vs COMBI.

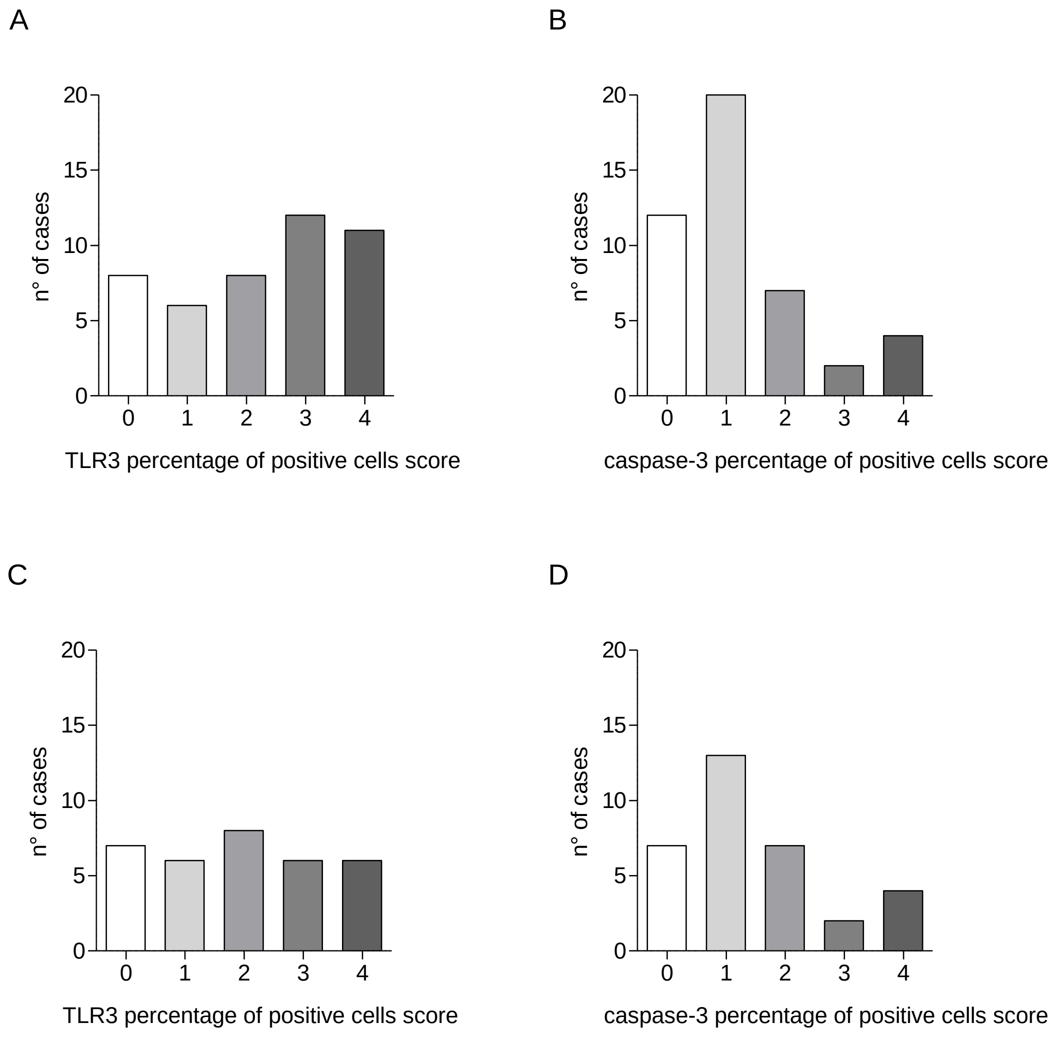

Supplementary Fig. 8. Distribution of NSCLC cases according to the percentage scores of TLR3 and caspase-3. TLR3 and caspase-3 expression on tumor cells were evaluated by the pathologist as percentage of positive cells out of the total number of immune cells within the sample (0; 1 ≤25%; 25 < 2 ≤50%; 50 < 3 ≤75%; 4 > 75%). A) TLR3 expression on tumor cells in the Niguarda cohort NSCLC cases; B) caspase-3 expression on tumor cells in the Niguarda cohort NSCLC cases; C) TLR3 expression on tumor cells in the adenocarcinoma cases of the Niguarda cohort; D) caspase-3 expression on tumor cells in the adenocarcinoma cases of the Niguarda cohort.

Supplementary Table 1. Clinical characteristics of NSCLC patients in Niguarda cohort.

	Overall cohort (N= 45)
Sex	
Female	15 (33%)
Male	30 (67%)
Age (median, yrs)	65,5 (range: 38-81)
Size	
<3	17 (38%)
=> 3	27 (60%)
NA	1 (2%)
Pathological status	
PT1	18 (40%)
PT2	18 (40%)
NA	9(20%)
Grade	
G1	1 (2%)
G2	15 (33%)
G3	19 (42%)
NA	10 (22%)
Lymph node metastasis	
N0	25 (56%)
N1	10 (22%)
NA	10 (22%)
Histological type	
Adeno	34 (76%)
Other	11 (24%)
